# Pentraxin 3 in Cardiovascular Disease

**DOI:** 10.3389/fimmu.2019.00823

**Published:** 2019-04-17

**Authors:** Giuseppe Ristagno, Francesca Fumagalli, Barbara Bottazzi, Alberto Mantovani, Davide Olivari, Deborah Novelli, Roberto Latini

**Affiliations:** ^1^Department of Cardiovascular Research, Mario Negri Institute for Pharmacological Research IRCCS, Milan, Italy; ^2^Humanitas Clinical and Research Center-IRCCS, Milan, Italy; ^3^Humanitas University, Milan, Italy; ^4^The William Harvey Research Institute, Queen Mary University of London, London, United Kingdom

**Keywords:** PTX 3, pentraxin, cardiovascular disease, cardiac arrest (CA), heart failure, biomarker

## Abstract

The long pentraxin PTX3 is a member of the pentraxin family produced locally by stromal and myeloid cells in response to proinflammatory signals and microbial moieties. The prototype of the pentraxin family is C reactive protein (CRP), a widely-used biomarker in human pathologies with an inflammatory or infectious origin. Data so far describe PTX3 as a multifunctional protein acting as a functional ancestor of antibodies and playing a regulatory role in inflammation. Cardiovascular disease (CVD) is a leading cause of mortality worldwide, and inflammation is crucial in promoting it. Data from animal models indicate that PTX3 can have cardioprotective and atheroprotective roles regulating inflammation. PTX3 has been investigated in several clinical settings as possible biomarker of CVD. Data collected so far indicate that PTX3 plasma levels rise rapidly in acute myocardial infarction, heart failure and cardiac arrest, reflecting the extent of tissue damage and predicting the risk of mortality.

## Introduction

According to the World Health Organization (WHO), an estimated 17 million people globally die of cardiovascular diseases (CVD) every year, with important implications in terms of quality of life and social costs^1^ Experimental and clinical evidence points to inflammation as a major cause of atherosclerosis, the underlying mechanism of CVD (1, 2). Accordingly, therapies targeting inflammation show promising results, as demonstrated by the successes of statins therapy, due not only to their effects on cholesterol, but also on the control of inflammation (3); or anti-interleukin-1β (IL-1β) appears to lower cardiovascular event rates (4).

The inflammatory response is mediated by a set of cells and soluble proteins belonging to the innate immune system. The humoral arm of the innate immune response includes components of the complement cascade and soluble pattern recognition molecules (PRM), particularly collectins (surfactant protein-A, [SP-A], and SP-D), ficolins, (ficolin-1;−2;−3) and members of the pentraxin family (C-reactive protein [CRP], serum amyloid P component [SAP], and long pentraxin 3 [PTX3]) (5–7). Therefore, soluble PRM are a heterogeneous group of proteins acting as functional ancestors of antibodies and key roles as regulators of inflammation playing as effectors and modulators of the innate immune response in animals and man.

CRP, one of the prototypic molecules of the pentraxin family, is a systemic biomarker of inflammation widely used in the clinic to monitor infections and inflammatory conditions (7). Epidemiological studies have consistently associated raised CRP serum levels with an increased risk of acute myocardial infarction (MI), stroke, and peripheral artery disease (8). In studies to date, CRP has emerged not only as a biomarker of CVD, but also as an independent predictor of adverse cardiovascular events.

**Figure 1:**
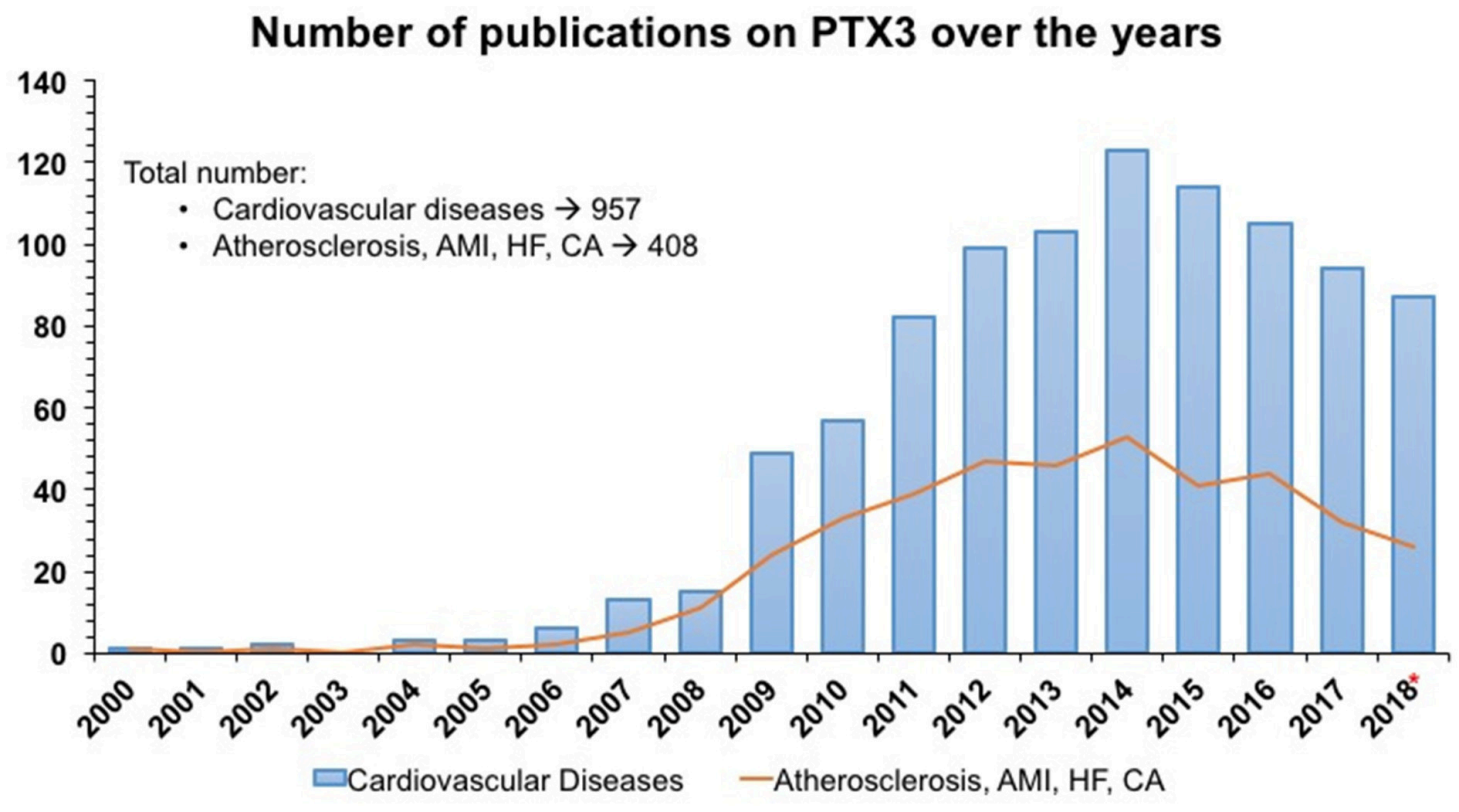
Number of publications/year from PubMed and Embase on PTX3 as an early indicator of acute myocardial infarction and cardiovascular diseases in humans. SEARCH QUERY: (ptx3 OR “pentraxin 3”/exp/ descriptor) AND “cardiovascular disease”/exp/descriptor AND 2000–2018*. Same search strategy was applied for PTX3 AND Atherosclerosis, Acute Myocardial Infarction (AMI), Heart Failure (HF) and cardiac arrest (CA). * 2018 included only 10 months.

PTX3, identified as a cognate molecule of CRP, is a multifunctional protein with complex regulatory roles in inflammation and extracellular matrix organization and remodeling (9). In men and mice PTX3 blood levels rise rapidly and dramatically in different pathological conditions with an inflammatory and/or infectious origin and have been investigated in several studies (Figure 1). The main characteristic of PTX3 is that it rises faster than CRP (peak at 6–8 h for PTX3; 24–48 h for CRP), very likely because of local vs. systemic production of the two proteins (9). The question is “How can a member of the humoral innate immunity be involved in cardiovascular health and disease?” Here we review the key properties of PTX3 as prototypic member of the pentraxin superfamily in relation to cardiovascular pathology.

The properties of PTX3 have been widely studied in humans and mice, using genetic approaches made possible by the high level of conservation of this molecule among species (5). The review will specifically deal with (1) vascular disorders, in which PTX3 has been found to play a role, but also (2) cardiac diseases such as myocardial infarction, heart failure (HF) and cardiac arrest (CA). While it has long been known that atherosclerosis is an inflammatory disease (10) and consequently innate and adaptive immune responses are expected to play a role, the involvement of PTX3 in cardiac diseases is somewhat less evident.

## The Pentraxin Superfamily: CRP and PTX3

The Pentraxin superfamily comprises long and short pentraxins (9). CRP and SAP were identified as the prototypes of the short pentraxin family; PTX3 was cloned in the late 1980's and is considered the prototype of the long pentraxin arm, its gene and protein sequences being almost twice the sequences of CRP and SAP. PTX3 is a key molecule playing complex regulatory roles at the crossroads of innate immunity, inflammation, tissue repair and cancer (9). A strong association has been reported between PTX3 genetic variants, affecting circulating levels of the protein, and susceptibility to fungal infections, suggests therapeutic use of the protein (11–15).

The main biochemical and biological characteristics of CRP and PTX3 have been amply described in several reviews, some of which published very recently (7, 9). Here we will only underline the main differences between the two proteins and some aspects possibly helpful to define their role in CVD.

**Figure 2:**
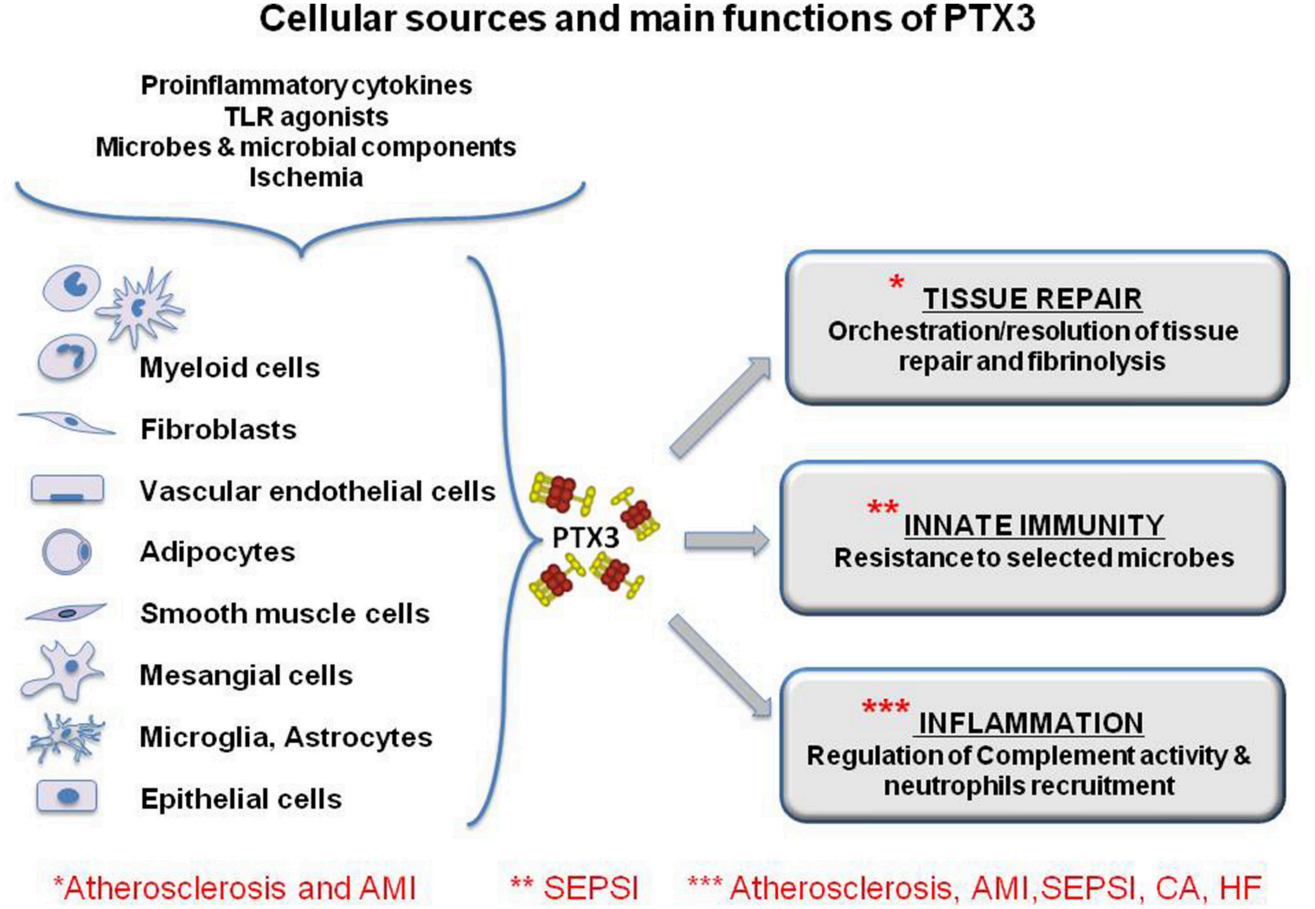
Cellular sources and main functions of the long pentraxin PTX3. The protein is induced by primary proinflammatory stimuli, TLR engagement, microbe recognition and ischemia by myeloid and stromal cells. PTX3 is a multifunctional protein playing a role in the orchestration of tissue repair through the regulation of fibrin deposition, and it regulates inflammation, modulating complement activity and limiting neutrophils infiltration in inflamed tissues. Finally, PTX3 is a molecule of the innate immunity and exerts protective roles against selected pathogens acting as and opsonin. The functional role of PTX3 in the main discussed pathology is highlighted by asterisks. However, for some of them, PTX3 has been explored only as a biomarker and there is little evidence of its functional involvement.

Although both CRP and PTX3 are considered acute phase proteins, they differ in their producing cells and inducing stimuli. The short pentraxins CRP and SAP are produced primarily in the liver in response to IL-6, reflecting a systemic response, while PTX3 is produced locally by a wide range of stromal and myeloid cells, including monocytes, endothelial cells (EC), and fibroblasts, but not hepatocytes (Figure 2). Primary pro-inflammatory signals, interleukin- (IL-) 1β (IL-1β) and TNFα, or bacterial moieties engaging Toll-like receptors (TLR), such as bacterial lipopolysaccharides (LPS), flagellin and outer membrane proteins, are major inducers of PTX3, while IL-6 is ineffective. Polymorphonuclear leucocytes (PMN) have a store of mature PTX3 produced during the differentiation from bone marrow precursors and accumulated in their granules, ready to be released in response to microbial recognition or tissue damage (16).

Vascular EC are a major source of PTX3 in response to inflammatory signals. Anti-inflammatory and atheroprotective signals, such as high density lipoproteins (HDL) and IL-10 induce PTX3 expression. This suggests a potential regulatory role of PTX3 in the innate and adaptive immune responses as well as being an anti-atherogenic molecule (17–19).

The NF-κB pathway is involved in the cascade of molecular events leading to PTX3 expression, as initially demonstrated in a model of acute myocardial ischemia (AMI) and reperfusion in mice (20), and subsequently confirmed by other studies (21, 22). In the model of myocardial infarction in mice, induction of ischemia resulted in upregulation of PTX3 production, an effect almost completely absent in *il-1r1-* or *myd88*-deficient mice.

Recent data have indicated a role of PTX3 in tissue remodeling and repair. In different models of tissue damage, PTX3 was localized in the pericellular provisional fibrin matrix, where it promoted migration and invasive phenotype of remodeling cells (23). Excessive fibrin accumulation was observed in skin, liver and lung injury models in *ptx3*-deficient mice, while in a murine model of arterial thrombosis PTX3 released by EC inhibited platelet aggregation, dampening thrombogenesis (23, 24). In addition, PTX3 is involved in edema resolution and scar formation in a model of brain ischemic injury in mice (25). Finally, we recently reported a non-redundant role of PTX3 in physiological skeletal remodeling and in proper matrix mineralization during bone fracture repair (26). These observations illustrated how PTX3 can play essential roles in tissue remodeling and repair.

The data summarized here indicate the complex regulation of PTX3 production from different cell types in response to different stimuli, and very likely reflect the different roles of this multifunctional protein in the innate immune response and as a constituent of the extracellular matrix. In addition, the induction of PTX3 by anti-inflammatory and atheroprotective signals such as HDL and IL-10 may reflect a possible protective function of PTX3 on EC and vascular integrity.

PTX3 is also expressed during sterile inflammation. For instance, in the model of experimental myocardial infarction (MI) mentioned, *ptx3*-deficient mice had greater myocardial lesions, more leukocyte infiltration, more cell death and higher complement C3 deposition in the infarcted area (20). This suggests that PTX3 might have a non-redundant cardioprotective role in mice, acting on the inflammation and tissue damage associated with reperfusion possibly by affecting the classical and the alternative pathways of complement activation (20). In addition, PTX3 can regulate leukocyte extravasation through an interaction with P-selectin (27), reducing neutrophil recruitment in inflamed sites. PTX3 can also interact with platelets via P-selectin exposed on their surface, and dampens the proinflammatory and prothrombotic effects of activated platelets, further contributing to a cardioprotective role (28).

In contrast, in a mouse model of transverse aortic constriction (TAC), PTX3 modulated the hypertrophic response and ventricular dysfunction following an increased afterload. Specifically, echocardiography indicated that PTX3 overexpression promoted tissue remodeling, left ventricular dysfunction, and increased myocardial fibrosis, while these responses were suppressed in *ptx3*-deficient mice (29).

## PTX3 in CVD

The findings summarized above underline the dual role of PTX3 in sterile and non-sterile inflammation. Here we will examine the role of PTX3 in four cardiovascular disorders:

1. Atherosclerosis,
2. Acute MI (AMI)
3. Heart failure (HF)
4. Cardiac arrest (CA).

We briefly discuss the evidences of the possible roles played by PTX3 and its potential as a circulating biomarker of diagnosis and/or prognosis in each disease.

### Atherosclerosis

PTX3 is produced by different cell types potentially involved in atherosclerosis, in particular EC, smooth muscle cells and macrophages (Figure 2). Staining of advanced atherosclerotic lesions in humans showed strong expression of PTX3, mainly by macrophages and EC, but also by smooth muscle cells (30). Different pro-inflammatory molecules are produced in an atherosclerotic lesion, particularly cytokines such as TNFα and IL-1, and oxidized low- density lipoproteins (oxLDL) These soluble factors may well be responsible for the production of PTX3 by target cells (31).

Investigations were made in PTX3/apolipoprotein E double knockout mice (*ptx3/apoE-/-*) The lack of PTX3 in animals with a genetic background making them susceptible to atherosclerosis resulted in larger areas of atherosclerotic lesions, greater accumulation of macrophages, higher expression of adhesion molecules, cytokines and chemokines in the vascular wall (19). Vascular inflammation was more marked, suggesting that PTX3 could exert an atheroprotective effect in mice.

Smooth muscle cells are important players in atherosclerosis and are activated after arterial injury. The soluble mediators produced by injured arteries include fibroblast growth factor 2 (FGF2), one of the well-characterized ligands recognized by PTX3 (32, 33). FGF2 plays important roles *in vivo* by promoting angiogenesis and revascularization during wound healing, inflammation, atherosclerosis, and tumor growth. PTX3 has been reported to act as a competitor of FGF2, blocking its interaction to its receptor and thus influencing neo-angiogenesis. In addition, it has been recently reported that PTX3 interaction with FGF2 might contribute to the maintenance of bone mass in homeostatic and pathological conditions, affecting the cross-talk between inflammatory cells and endothelium (26, 34). The specific interaction between PTX3 and FGF2 also results in the inhibition of FGF-dependent proliferation *in vitro* (35). In addition, FGF2 exerts a potent inhibitory effect on the activation of smooth muscle cells, suggesting that PTX3 might affect the activation of SMC after arterial injury (35).

In summary, there are various evidences that PTX3 may play a role in atherosclerosis:

1. PTX3 is expressed more in leukocytes and in adipose tissue from patients with high levels of low-density lipoprotein (LDL) compared to those with low levels (36).
2. PTX3 expression in visceral fat of obese individuals is determined by both LDL/ high density lipoprotein (HDL) ratio and fibrinogen (37).
3. Treatment of EC with lysophosphatidic acid led to a marked upregulation of PTX3 both in terms of mRNA and protein level (38).
4. Immunohistochemistry (ICH) on human atherosclerotic lesions showed that macrophages and PMN cells infiltrating the atherosclerotic plaques were positive for PTX3 (30, 39).
5. PTX3 expression in human EC was upregulated by HDL, whereas there were no effects on CRP and SAP expression (19).
6. In *apolipoprotein E*-deficient mice, the inflammatory reaction of the vascular wall and macrophage accumulation in the plaque were markedly increased by the lack of PTX3 (19, 31).
7. PTX3 plays a protective role in arterial thrombosis by dampening the pro-thrombotic effects of fibrinogen and collagen (23, 24)

### Acute Myocardial Infarction (AMI)

One of the first *in vivo* findings on PTX3 was its high expression in murine hearts after injection of LPS (40). Specific immunostaining for PTX3 was also observed in heart tissues of patients who died of MI (41). In early ischemic lesions PTX3 expression was high primarily in PMN cells, while in more advanced lesions PTX3 positivty of granulocyte was lost and was mainly acquired by macrophages, EC and sometimes myocardiocytes (Figure 2).

The high conservation of PTX3 in evolution allows us to translate to humans the observations in mice, whereas CRP and SAP expression is regulated differently in mice and man. Based on this consideration, a model of experimental MI based on coronary artery ligation and reperfusion was applied to *ptx3*-deficient mice (20). In this model, PTX3 mRNA expression was upregulated in the left ventricle (LV) of wild- type animals and circulating levels of the protein were increased, with a peak at 24 h. IHC and confocal microscopy confirmed that major sources of PTX3 in the infarcted heart are first granulocytes and EC (24 h after reperfusion), followed by macrophages, that became positive 3 days after reperfusion. Similarly, PTX3 is released from neutrophils in the early phases of AMI in humans (28), contributing to the rapid increases in different studies (see below). Infarct sizes were measured in wild type and *ptx3-*deficient mice, and the larger damaged area was in the absence of the protein (20). Thus, the presence of PTX3 observed by IHC in tissue samples from mice after ischemic injury and confirmed in the heart of patients who died from MI, supports a pathophysiologic role of the protein in myocardial damage and repair.

Regulation of complement activation by PTX3 has been considered a possible mechanism involved in tissue damage after ischemia and reperfusion. The interaction of PTX3 with Factor H (FH), the most important regulator of the alternative complement pathway, was important to limit FH deposition on PTX3-coated surfaces and to protect against oxidative stress-induced complement and inflammasome activation (42, 43). In addition, deposition of FH and higher complement activation was lower in tumors growing in *ptx3-*deficient mice (44). These observations strongly sustain the hypothesis that the PTX3-FH interaction may constitute a mechanism to prevent excessive complement activation. In the infarcted heart, C3 deposition was higher in *ptx3*-deficient mice and complement depletion canceled the difference between wild- type and *ptx3*^−/−^ mice (20).

Whether PTX3 plays a role in the progression of post-infarction left ventricular dysfunction and failure has been the subject of research in the mouse after coronary ligation. The results have been mostly inconclusive but nonetheless it can be concluded with some confidence that the role of PTX3 in left ventricular remodeling after MI is practically irrelevant.

**Figure 3:**
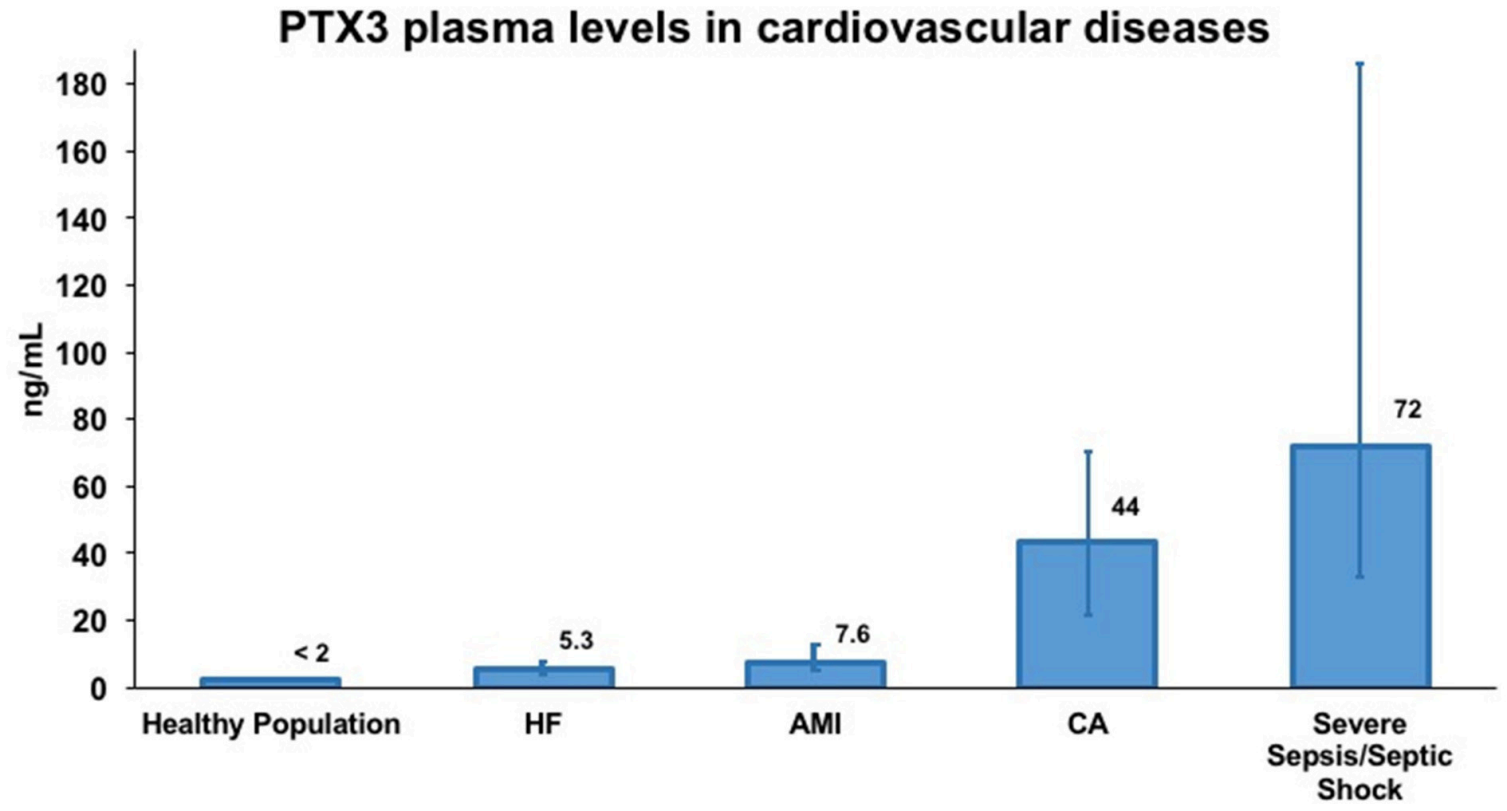
Representative concentrations of PTX3 in healthy volunteers and in different cardiovascular diseases. Data were extrapolated from four different publications evaluating PTX3 levels in plasma samples by sandwich ELISA. Specifically, PTX3 levels in healthy volunteers and in acute myocardial infarction (AMI) patients are from Peri et al. (53) in heart failure (HF) patients are from Latini et al. (56) in cardiac arrest (CA) patients are from Ristagno et al. (57) and in severe sepsis/septic shock patients are from Caironi et al. (58). PTX3 levels are presented as median (interquartile range). Numbers over bars represents the median value of plasma concentration of PTX3 expressed in ng/mL.

The production of PTX3 by vascular cells in response to inflammatory signals and ox-LDL (17, 31) and its occurrence in atherosclerotic lesions (30, 45), prompted investigations of PTX3 levels in AMI (46–52). A high- sensitive (lower detection limit 0.1 ng/mL), specific (no cross-reaction with human CRP and SAP) ELISA based on original reagents was developed and used to measure circulating levels of PTX3 in patients and healthy volunteers. Plasma PTX3 in healthy subjects was ≤ 2 ng/mL, with higher levels in females than males and levels increased with aging (53–55). Patients with AMI showed an early peak of PTX3 plasma levels observed within 6–8 h from symptom onset (Figure 3), and baseline levels were reached within 3 days (53, 59). In a cohort of 748 patients with MI and ST elevation enrolled in the Lipid Assessment Trial Italian Network (LATIN), PTX3, CRP, pro b-type natriuretic peptide (NT-proBNP) and troponin-T were measured within the first day from the onset of symptoms. Among all these markers, PTX3 levels >10.73 ng/mL within the first day after MI were the only independent predictor of three-month mortality (56). High PTX3 also predicted long-term mortality in several subsequent prospective observational studies (50–52).

Besides being a biomarker of MI reflecting the degree of tissue damage, PTX3 was proposed as a prognostic tool in two large studies aiming to identify predictive factors of CVD: the Cardiovascular Health Study (1,583 patients analyzed) and the Multi-Ethnic Study of Atherosclerosis (2,880 patients). These two studies illustrated a significant relation between PTX3 levels and cardiovascular mortality and all-cause death (60, 61). In addition, it was recently shown that higher PTX3 levels predicted occurrence of MI in a cohort of young or middle-aged individuals followed for a first-time MI (62).

It is not clear yet whether the impressive relation with fatal outcome seen in most of the clinical studies actually reflects a role of PTX3 on the pathogenesis of damage, for instance through amplification of the complement and coagulation cascades (63, 64), or a marked protective response to severe cardiac injury. This question has not yet been addressed, nor has that more related to the potential role of PTX3 as an early prognostic biomarker in MI.

### Heart Failure (HF)

The role of inflammation in the progression and outcome of heart failure (HF) is still under discussion. High levels of circulating inflammatory molecules, in particular cytokines and CRP, are related to more severe HF and worse outcomes. However, whether inflammation is a cause or just a consequence of the disease is a matter of controversy. In addition, none of the inflammatory cytokines measurable in the plasma of HF patients can be used singly as a basis for prognosis (65), or even when a multi-marker approach including a range of soluble inflammatory mediators and PTX3, was considered (66).

The prognostic role of PTX3 in chronic HF with reduced (67, 68) or preserved (69, 70) ejection fraction has been reported in several small studies (with ≤ 200 patients each). PTX3 levels correlated weakly with those of brain natriuretic peptide (BNP), and ROC analysis suggested that PTX3 was superior to BNP in the prediction of adverse outcomes (68). Other studies (with 37–164 patients) showed that the best risk prediction was achieved by combining three biomarkers: BNP, H-FABP and PTX3 (67, 71). Importantly, high levels of PTX3 correlated significantly with the presence of HF among patients with normal LVEF and LV diastolic dysfunction (69). PTX3 was assayed at randomization and after 3 months in 1,233 patients from the GISSI-Heart Failure trial (GISSI-HF) and 1,457 patients from the Controlled Rosuvastatin Multinational Trial in HF (CORONA) (72). PTX3 was independently and significantly related to the severity of HF. In addition, PTX3 levels were higher in older individuals with ventricular dysfunction, worse symptoms and co-morbidity, i.e., atrial fibrillation or diabetes. Most important, baseline concentrations of PTX3 and three-month changes were significantly related to fatal outcome (72). Similarly, a long-term prospective study on patients with HF and normal ejection fraction indicated that high baseline circulating levels of PTX3 were predictive of all-cause mortality, cardiovascular mortality or hospitalization for worsening HF (70).

The effects of rosuvastatin in the GISSI-HF and CORONA studies deserve a comment. Rosuvastatin (10 mg/day for 3 months) consistently reduced circulating levels of high-sensitive CRP (hsCRP) in both the CORONA trial and GISSI-HF, in line with its anti-inflammatory properties. In contrast, the CORONA trial found an unexpected, opposite effect on PTX3, which increased significantly more in rosuvastatin- treated patients than with placebo (72). This controversial observation might be explained by the hypothesis that statins alter the innate immunity behavior of stimulated phagocytic cells and enhance the production of macrophage and neutrophil extracellular traps that contain antimicrobial proteins and PTX3 (16, 73). This hypothesis, based on clinical and epidemiological observations of lower susceptibility to severe bacterial infection in patients receiving statin therapy, remains highly speculative but calls for further investigation. According to these findings CRP and PTX3 seem to exert different but, to a certain extent, overlapping roles in systemic and local inflammation.

### Cardiac Arrest (CA)

Despite the return of spontaneous circulation (ROSC), mortality after resuscitation from cardiac arrest remains extremely high (74). The well-known “post-cardiac arrest syndrome” (PCAS) is characterized by myocardial dysfunction with circulatory shock, systemic inflammation, and evolving brain injury (75). Thus, clear similarities between sepsis, septic shock and PCAS have been acknowledged (76). Indeed, upon reperfusion after ROSC, a systemic inflammatory response occurs and ultimately contributes to worsening of circulatory shock and neurological damage.

Recently PTX3 has been investigated in comparison with the classic hsCRP for the prediction of early multiple organ dysfunction syndrome (MODS), early death, and long-term outcome after CA. More specifically, PTX3 and hsCRP were assayed at admission to intensive care unit (ICU) and 2 days later in 278 out-of-hospital CA patients enrolled in the prospective observational cohort study FINNRESUSCI, conducted in 21 hospitals in Finland (57). In this population, at ICU admission hsCRP was normal, i.e., 2.8 [1.2–9.8] mg/L, while PTX3 already showed large increases, i.e., 19.1 [9.2–41.8] ng/mL and levels were higher in older patients and in patients resuscitated after longer CA. Higher plasma levels of PTX3 were significantly associated with MODS [AUCs 0.78 (*p* < 0.0001)], and values above 24 ng/mL showed 0.8 sensitivity and 0.7 specificity for predicting MODS. HsCRP, instead, presented a lower accuracy (AUC of 0.6, *p* = 0.033) compared to PTX3 (*p* < 0.003) in predicting MODS occurrence. PTX3 plasma levels were already significantly higher at ICU admission in patients who developed MODS and died in the ICU compared to those who did not experience MODS and survived to ICU discharge. HsCRP levels discriminated for MODS and ICU death only 48 h after admission (57).

After ROSC, the levels of soluble intercellular adhesion molecule-1, soluble vascular-cell adhesion molecule-1, and P- and E-selectins showed early increases indicating leucocyte and endothelial activation. This condition is ultimately associated with a rapid PTX3 increase, as illustrated by the 10-fold higher plasma levels already observed at ICU admission in the cohort study. Thus, early PTX3 levels, i.e., at ICU admission after ROSC, are independent predictors of MODS and early death, while CRP is not. Moreover, since PTX3 levels continue to rise during ICU recovery, the post-CA pro-inflammatory response is prolonged and could be therefore a potential target for intervention (57).

### PTX3 as Circulating Biomarker in CVD

Preclinical data in the mouse and the homology with CRP, a molecule used to monitor inflammatory diseases and infection in clinical practice, prompted investigation of PTX3 as possible marker of human pathology. As seen above, PTX3 has been investigated as a possible circulating biomarker in MI, HF and CA, but the numbers of pathological conditions potentially involving PTX3 as biomarker are larger. The rapid rises in PTX3 plasma levels are compatible with an acute phase response. PTX3 blood levels can reach 800 ng/ml in patients with endotoxin shock, sepsis and infections of viral, bacterial or fungal origin (58, 77–83). In general, PTX3 circulating levels were significantly correlated with the severity of disease and mortality, and served to monitor the response to therapy. In addition, PTX3 levels rarely correlate with CRP, indicating that the two proteins might have different roles. This lack of correlation was useful to distinguish the presence and absence of shock in a small cohort of patients with meningococcal disease (80). In addition, myeloproliferative disorder patients with major thrombosis had higher levels of hsCRP and lower levels of PTX3, thus confirming that the two molecules modulate cardiovascular risk factors in opposite ways (84).

The diagnostic and prognostic value of circulating PTX3 was tested in recent studies on patients with severe sepsis and septic shock. The Albumin Italian Outcome Sepsis (ALBIOS) trial reported that high levels of PTX3 measured in a cohort of 958 patients on day 1 after admission to the ICU were able to predict new organ failures, while a smaller drop in circulating PTX3 over time predicted an increased risk of death (58). Similar increases in plasmatic levels of PTX3 and associations with mortality in patients with sepsis or septic shock were reported in other studies (85–88).

PTX3 plasma levels were significantly elevated in patients with arterial inflammation who underwent percutaneous coronary intervention (PCI) (54, 89, 90). Systemic PTX3 levels before PCI were associated with larger plaque area and volume, a higher risk of plaque rupture at the culprit site and impaired post-PCI myocardial perfusion (49). A study on 594 patients with stable coronary artery disease (CAD) reported that PTX3 plasma levels were higher 24 h after PCI than before (91). During the follow-up for major adverse cardiovascular events, patients with higher post-PCI levels of PTX3 had a higher incidence of events. Similarly, in patients with angina who underwent PCI, PTX3 levels resulted to be an independent risk factor associated to troponin increase after PCI (92). These data suggest that PTX3 could provide a reliable marker for risk stratification in patients undergoing PCI. Patients with unstable angina pectoris have higher PTX3 levels than healthy controls, suggesting that this long pentraxin might be a candidate marker to unstable angina (54).

PTX3 has also been proposed as a novel marker for stent-induced inflammation in patients with CAD after PCI (93). PTX3 was increased in peripheral blood and in the coronary sinus of 20 patients undergoing coronary stenting. Expression of CD11b/CD18 on neutrophils correlated with PTX3 levels. The relative PTX3 increase observed at 24 h was the most powerful predictor of late lumen loss. In the same setting, CRP could not discriminate between patients with and without re-stenosis. These data suggested that, after vascular injury PTX3 may be used as a marker of the inflammatory response and neointimal thickening. Patients with re-stenosis after PCI presented a positive transcardiac gradient, indicative of PTX3 production by the coronary vasculature (93). The role of PTX3 in patients with CAD was confirmed in a subsequent prospective observational study on 75 ST elevation MI patients. High levels of PTX3 before PCI were associated with higher frequencies of plaque rupture (49).

PTX3 levels were high in patients with small vessel vasculitis and rheumatoid arthritis, but not in those with systemic lupus erythematosus (94, 95). In small vessel vasculitis, a group of autoimmune disorders characterized by inflammation of the blood vessels, PTX3 plasma levels were higher in patients with active disease than in those with quiescent disease (95). EC are responsible for PTX3 production, as shown by IHC performed on skin sections at sites of vasculitis (96). Moreover, PTX3 is more abundant at sites of leukocytoclastic infiltration (97). PTX3, in contrast to the short pentraxin SAP, inhibits the uptake of apoptotic PMNs by macrophages (97), suggesting that the long pentraxin is a key factor in the incomplete clearance of apoptotic and secondary necrotic PMNs observed in small-vessels vasculitis (96). High circulating PTX3 levels were associated with vascular injury in systemic lupus erythematosus patients, thus increasing the dysfunction on the vascular endothelium (98).

PTX3 plasma levels also increase in patients with chronic kidney disease (CKD) (99), and correlate with the severity of the disease (100, 101). Hemodialysis (HD) patients have higher circulating levels of PTX3 compared to peritoneal hemodialysis patients (102). During the HD session, PTX3 plasma levels are increased, suggesting that the protein could be a biomarker of the HD-induced inflammation (103). In addition, in the presence of peripheral or coronary artery disease, PTX3 levels are significantly increased (102). Finally, high PTX3 levels predict all-cause mortality and cardiovascular mortality in patients with CKD (99), a finding reminiscent of MI data (56). PTX3 was associated with proteinuria and endothelial dysfunction in patients with advanced CKD or type 2 diabetes (104), suggesting that PTX3 is more than just an additional marker of inflammation in chronic HF (105).

Preeclampsia, a pathological condition causing an exaggerated inflammatory response resulting in endothelial dysfunction (106–108), is a major complication of pregnancy. Circulating levels of PTX3 are high in preeclampsia, underlining the strong inflammatory response (108, 109). In addition, PTX3 levels in the first trimester were altered in women who subsequently developed preeclampsia, this confirming that an excessive inflammatory response is one of the causal factors causing preeclampsia in pregnant women (110).

## Concluding Remarks

PTX3, the prototype member of the long pentraxin family, is a soluble pattern recognition molecule with multifunctional properties. Genetic approaches indicate that PTX3 is an essential component of innate immunity and a modulator of the inflammatory response. Not surprisingly in an intricate field such as the immune/inflammatory response, PTX3 has a dual character, one is good-protective against excessive inflammatory response, the other is harmful- antiangiogenic in cardiovascular diseases or inhibitor of phagocytosis in nasopharyngeal carcinoma (111). In addition, the recent observations on the involvement of PTX3 in tissue remodeling and repair (23–26) may cast further light on the role of this molecule in the cardiovascular pathology.

Current data are consistent with a role of PTX3 as a novel marker of CVD (Figure 3). In general, PTX3 levels rise rapidly, reflecting the inflammatory response affecting vascular involvement. Thus, PTX3 has a different kinetics of production and different patterns of recognized ligands from CRP, a much more widely used biomarker of inflammation and infection. Data available so far propose that CRP and PTX3 could serve as complementary biomarkers of pathological conditions, with CRP reflecting a systemic response while PTX3 is mainly produced locally. However, one must acknowledge that, while the functional role of PTX3 in some vascular disorders, i.e., atherosclerosis and MI, has been well-described, in HF and CA, PTX3 has been explored only as a biomarker and there is little evidence of its functional involvement. In fact, the crucial question on each candidate biomarker is “How long can a biomarker be called “emerging”? Are 10 years enough? Or better 20? How long should we keep searching for evidence? It is impossible to answer this rationallly. Although almost 50 years passed since the discovery of natriuretic peptides and their functions, the evidence of benefits obtained from their clinical monitoring is still incomplete.

## Author Contributions

GR, BB, and RL equally contributed to work conception and draft manuscript. FF, DO, and DN contributed to literature review and draft parts of the manuscript. AM contributed to manuscript revision.

### Conflict of Interest Statement

The authors declare that the research was conducted in the absence of any commercial or financial relationships that could be construed as a potential conflict of interest. The reviewer LD declared a past co-authorship with several of the authors to the handling editor.

## Footnotes

^1^Source: http://www.euro.who.int/en/health-topics/noncommunicable-diseases/cardiovascular-diseases/cardiovascular-diseases2.
